# Mortality and Length of Stay of Very Low Birth Weight and Very Preterm Infants: A EuroHOPE Study

**DOI:** 10.1371/journal.pone.0131685

**Published:** 2015-06-29

**Authors:** Dino Numerato, Giovanni Fattore, Fabrizio Tediosi, Rinaldo Zanini, Mikko Peltola, Helen Banks, Péter Mihalicza, Liisa Lehtonen, Sofia Sveréus, Richard Heijink, Søren Toksvig Klitkou, Eilidh Fletcher, Amber van der Heijden, Fredrik Lundberg, Eelco Over, Unto Häkkinen, Timo T. Seppälä

**Affiliations:** 1 Centre for Research on Health and Social Care Management, Bocconi University, Milan, Italy; 2 Dipartimento Materno Infantile, Ospedale "A Manzoni", Lecco, Italy; 3 Centre for Health and Social Economics, National Institute for Health and Welfare, Helsinki, Finland; 4 Semmelweis University, Budapest, Hungary; 5 Turku University Hospital and Turku University, Turku, Finland; 6 Medical Management Centre, Karolinska Institutet, Stockholm, Sweden; 7 National Institute for Public Health and the Environment, Bilthoven, the Netherlands; 8 Department of Health Management and Health Economics, Institute of Health and Society, University of Oslo, Oslo, Norway; 9 Lothian Analytical Services, NHS Lothian, Edinburgh, Scotland; 10 The EMGO Institute for Health and Care Research, VU University Medical Center, Amsterdam, the Netherlands; 11 Department of Neonatology, Linköping University Hospital, Linköping, Sweden; Centre Hospitalier Universitaire Vaudois, FRANCE

## Abstract

The objective of this paper was to compare health outcomes and hospital care use of very low birth weight (VLBW), and very preterm (VLGA) infants in seven European countries. Analysis was performed on linkable patient-level registry data from seven European countries between 2006 and 2008 (Finland, Hungary, Italy (the Province of Rome), the Netherlands, Norway, Scotland, and Sweden). Mortality and length of stay (LoS) were adjusted for differences in gestational age (GA), sex, intrauterine growth, Apgar score at five minutes, parity and multiple births. The analysis included 16,087 infants. Both the 30-day and one-year adjusted mortality rates were lowest in the Nordic countries (Finland, Sweden and Norway) and Scotland and highest in Hungary and the Netherlands. For survivors, the adjusted average LoS during the first year of life ranged from 56 days in the Netherlands and Scotland to 81 days in Hungary. There were large differences between European countries in mortality rates and LoS in VLBW and VLGA infants. Substantial data linkage problems were observed in most countries due to inadequate identification procedures at birth, which limit data validity and should be addressed by policy makers across Europe.

## Introduction

The rate of preterm births is increasing worldwide and great disparities exist in survival rates and quality and access to care across countries, particularly regarding very low birth weight (VLBW) infants, less than 1500 grams at birth, and very low gestational age (VLGA) infants, less than 32 weeks [1,2]. Although it is assumed that there are country variations in performance and outcomes for VLBW and VLGA infants [3–5], most population-based studies have focused on a single country [1,6], on single regions in different countries [7] or on differences between two countries [3]. Several national [3,6,8–10] and international [11] neonatal networks have been established to routinely gather and analyse data on VLBW and VLGA infants to provide comparison data for quality surveillance and factors influencing mortality and morbidity. However, they commonly assess a selective sample of infants treated in neonatal intensive care units (NICU) that are recognized for excellence and participating in the networks. As a consequence, these comparative studies have a limited capacity to capture the performance patterns of the overall organization of care for all VLBW and VLGA infants and to address enduring disparities in treatment due to regional and national differences.

The EuroHOPE (European Health Care Outcomes, Performance and Efficiency) study uses administrative data that has a more comprehensive perspective since it potentially includes all neonatal care, extending the focus of interest beyond “excellent” hospital structures as well as beyond the first hospitalization. Furthermore, the EuroHOPE study provides a multi-country international comparison of a vulnerable and growing segment of the population. Finally, it provides a shared methodology for standardising widely available administrative data from various countries in such a way that allows for regular population-based monitoring in the future as an ongoing international comparison [12], without the need for costly survey research, center enrolment or manual consultation of hospital records.

The aim of this study is to compare mortality rates and hospital care use of VLBW and VLGA infants in seven European countries; a second objective is to provide some insight regarding the validity and feasibility of using an administrative database approach for this population, comparing incidence and outcomes measured in the EuroHOPE study to those reported in the literature and providing some recommendations for how to improve data collection, preparation and use.

## Materials and Methods

### Study population

The study population consisted of VLBW and VLGA infants born between 1st Jan 2006 and 31st Dec 2008 in seven European countries (Finland, Hungary, Italy (the Province of Rome), the Netherlands, Norway, Scotland, and Sweden). Due to limited data availability, only infants born between 2008 and 2009 in Norway, and between 2006 and 2007 in the Netherlands, were analysed. Italian data is decentralized, managed autonomously by the different regions. Due to privacy regulations and the quality of the regional Medical birth registries (MBR), of three potential areas surveyed for data suitability, only the Province of Rome provided a sufficiently high data linkage rate with the MBR for the study. Infants were followed for up to 365 days after the day of birth; stillborn infants were not included in the study population. The live-born infants selected for cohorts complied with at least one of the two criteria for inclusion: weight at birth less than or equal to 1500 grams and gestational age (GA) less than 32 weeks. In other words, all infants below or equal to 1500 g and all infants below 32 weeks were included. Infants with incorrect identification numbers (ID) were excluded as this precluded linkage between records from Medical birth registry and other information registries. Infants with the following lethal congenital malformations were also excluded (ICD-9 and ICD-10 codes, respectively, are listed after each): anencephaly (740.0, Q00.0), transposition of great vessels (745.15, Q20.1), hypoplastic left heart syndrome (746.7, Q23.4), renal agenesis and dysgenesis (753.0, Q60.2), anomalies of diaphragm (756.6, Q79.0, Q79.1), Patau's syndrome (758.1, Q91.7), and Edward's syndrome (758.2, Q91.3).

Infants born before 22 weeks of GA and after 39 weeks of GA were excluded, as were infants with major disparities between GA and weight. Major disparities between GA and weight were defined to exclude likely transcription errors in the registries—a detailed description of the methods appears in the Discussion Paper, available at www.eurohope.info. Finally, in each country the infants with a length of stay (LoS) for all initial (continuous) hospital treatment longer than the 99th percentile in that country were excluded. Presumably a very long LoS could reflect errors in data or very severely ill infants not captured properly with our exclusion criteria which could compromise comparison of countries.

In each country, the MBR was used as the primary source of information, and subsequently linked with the hospital discharge register (HDR) and the Causes-of-Death registries. A Causes of Death registry does not exist in Hungary; however, the social security ID registry records deaths without reporting the ICD code for the cause. The registries were linked at the individual patient level using national personal ID, except in Hungary and the Netherlands, where deterministic and stochastic linkage were employed since the MBR and/or HDR does not include an ID for the newborn. Infants in other countries with a missing or incomplete ID in the MBR were excluded from the study. In Sweden and Norway, information on mortality was linkable with information on birth; however, difficulty linking the MBR to the HDR meant that follow-up treatment through one year was available for only 58% and 65%, respectively, of infants. This limitation was nationwide and not related to some specific regions; analysis revealed that there was a bias toward the exclusion of infants with a poorer prognosis that would have downwardly skewed mortality rates. In order to increase the comparability of the data, the larger population of VLBW and VLGA infants identified from birth records in Sweden and Norway was used to calculate mortality figs, even though the information on their treatment during the first year of follow-up was incomplete. Consequently, however, the figs for the LoS for both of these countries cannot be considered representative. They are presented but refer to the restricted samples of infants for whom linkable information about follow up treatment was available. In Scotland a similar problem is believed to have occurred where the medical birth record for between 10 to 15% of infants meeting the criteria could not be linked to mortality and hospital information. It is hypothesized that these infants, like in Sweden and Norway, had a poorer prognosis and that this has led to a bias towards a relatively healthier cohort for this study in relation to published reports [13]. In Italy and Hungary problems with linkage were related to the delayed assignment of ID for newborns. In the Netherlands, problems included data availability only for an earlier study period (2005–2007) in a time of improving mortality rates, a low linkage rate (estimated to be about 72%) and the disproportionate exclusion of multiple births, particularly same-sex multiple births, because of ID number difficulties, all of which likely affected mortality outcomes. In addition, national guidelines at the time recommended active treatment only for infants > = 25 weeks GA and abortion is legal up to 24 weeks GA, which are both assumed to have worsened mortality rates for infants <25 weeks GA.

For each infant, the first hospital episode (FHE) was defined to include all continuous hospital stays, including transfers, from the day of birth (index day) to the day of discharge to home from the last continuous hospital stay, or death. Subsequent episodes of hospital care were used to calculate the total number of days spend in hospital in the first year of life.

### Ethics

This study was approved by the commissioner of the research, the European Commission. The use of data was also approved by the data providers as follows: National Institute for Health and Welfare (Helsinki, Finland), the National Institute for Quality- and Organizational Development in Healthcare and Medicines (Hungary) and the Health Insurance Fund Administration (Hungary), Department of Epidemiology of the Regional Health Service—Lazio (Italy), the Statistics Netherlands and the Netherlands Perinatal Registry (the Netherlands), the Medical Birth Registry of Norway, Statistics Norway (Norway), the Information Services Division of National Services Scotland, NHS (Scotland), and the Swedish National Board of Health and Welfare (Sweden). The analysis was performed on anonymized and de-identified data. The conditions restricting the use of the data are presented in S1 Table.

### Statistical analysis

To enhance the comparability of the outcome measures, the outcomes were adjusted for GA (categorized; <25 weeks, 25–26 weeks, 27–28 weeks, 29–30 weeks, 31–32 weeks, >32 weeks), sex, intrauterine growth (defined in reference to Fenton’s [14] standardized international values), Apgar score at five minutes, parity and multiple births. A risk adjustment approach of dividing the observed number of outcomes by the expected number was applied (see e.g. Ash et al. [15]). These ratios were then multiplied by the average of the outcome measure in the pooled data of Finland, Hungary and Italy to get the risk adjusted mortality percentage or the risk adjusted LoS. In our study, the number of expected outcomes was based on modelling with logistic regression for mortality, and with negative binomial regression for LoS. [16] First, the regression models were estimated using pooled individual-level data from Finland, Hungary and Italy to get coefficients for the risk adjustment factors, separately for each outcome. Then, to acquire the expected number of outcomes in a country, each partner applied in their national data the estimated pooled coefficients for the factors included in the risk adjustment (using Stata version 12).

## Results

In the seven countries, 16,087 VLBW and VLGA newborn infants were included in the EuroHOPE dataset; after exclusions 15,373 (95.6%) remained and were used for the mortality analysis. For the LoS analysis, an additional 436 infants in Norway and 1,325 infants in Sweden were excluded because it was not possible to link the HDR to the MBR to measure follow-up, as described above. The proportion of VLBW and VLGA infants over all live-born infants in the EuroHOPE datasets varied between 0.76% in the Netherlands and 1.21% in Hungary, and are presented in Table 1, along with the comparison to surveillance data [17].

**Table 1:** Number and proportion of VLBW and VLGA infants among live-born infants in EuroHOPE data.

	Finland	Hungary	Italy	Netherlands^a^	Norway^a^	Scotland	Sweden
Total number of VLBW and VLGA infants born in 2006–2008	1584	3702	1382	4096	1428	2451	3229
VLBW and VLGA infants after exclusions	1455	3562	1265	2628	1311	2015	3137
Linkage rate (%): possibility to link the MBR with the HDR	99%	76%	87%	65%	100%^**c**^	85%	100%^**d**^
EuroHOPE proportion, %, of VLBW and VLGA infants among live-born infants (proportion for length of stay analysis)	0.82	1.21	1.04	0.76	1.22	1.16	0.97
Euro-Peristat 2008 [17],^b^ incidence, %, <1500g	0.8	1.4	0.8	1.0	0.9	1.1	0.7
Euro-Peristat 2008 [17],^b^ incidence, %, <32 weeks GA	0.9	1.4	1.0	1.1	1.0	1.2	0.9

^a^The Netherlands: 2006–2007, Norway: 2008–2009

^b^The European Perinatal Health Report 2008 is based on data from 2004, incidence figs are per 100 live-born infants

^c^The linkage is between MBR and Cause of Death Registry. The linkage with the HDR for LoS analysis was 65%

^d^The linkage is between MBR and Cause of Death Registry. The linkage with the HDR for LoS analysis was 58%

The baseline characteristics of the infants, their mothers and information on delivery are shown in Table 2. A supporting table (S2 Table) also shows the differences in the two datasets for Norway and Sweden, one for mortality (all) and one for follow-up care for measuring LoS (linkable). For Scotland, two columns also illustrate the two sets of data: the information from the MBR (all) and the (linkable) dataset (MBR records linked to information on mortality and hospital discharges) covering approx. 85% of all infants listed in the MBR and believed to represent a relatively healthier population. Distribution of infants across GA and birthweight groups is presented in a supporting table (S3 Table).

**Table 2:** Characteristics of VLBW and VLGA infants and mothers and overall unadjusted mortality in the EuroHOPE datasets in seven European countries.

	Finland	Hungary	Italy	Netherlands	Norway	Scotland	Sweden
Number of VLBW and VLGA infants born in 2006–2008^a^	1584	3702	1380	4096	1428	2451	3229
Number of VLBW and VLGA infants, after exclusions	1455	3562	1265	2628	1311	2330	3137
Mean (SD) gestational age, in weeks	28.9 (2.8)	29.1 (3.0)	29.5 (2.8)	29.7 (2.5)	29.0 (2.7)	29.3 (2.8)	28.8 (2.7)
Birth weight, in grams, mean (SD)	1215 (392)	1209 (400)	1243 (407)	1277 (362)	1227 (391)	1254 (372)	1247 (414)
Female gender (%)	45.3	49.1	46.2	44.9	45.2	46.4	44.5
Apgar score at five minutes, median	8	8	7	9	8	9	8
Appropriate for gestational age (%)	78.2	71.5	67.7	72.6	77.9	76.4	80.3
Small for gestational age (%)	4.8	5.9	6.7	4.8	5.3	6.0	6.0
Multiple birth (%)	33.5	29.1	32.9	14.2	27.2	26.7	27.8
First delivery (%)	52.4	51.5	66.7	62.0	53.8	58.3	83.5
Ceasarean delivery (%)	67.3	70.2	77.8	53.6	64.5	57.4	66.3
Malformations, number (%)	45 (3.1)	9 (0.3)	196 (15.5)	21 (0.8)	117 (2.9)		182 (5.8)
Mother’s characteristics, age in years (SD)	30.7 (5.9)	30.0 (5.7)	33.2 (5.9)	30.1 (5.2)	30.8 (5.8)	28.8 (6.6)	31.2 (5.6)
Mothers over 34 years (%)	79.4	19.6	42.4	20.6	27.4	22.5	26.1
Unadjusted 30 day mortality (%)	11.4	15.7	12.7	11.5	9.7	NA	8.0
Unadjusted 1 year mortality (%)	12.9	18.1	13.8	13.1	11.2	NA	9.4

^a^ 2008–2009 for Norway, 2005–2007 for Netherlands.

The mean values of birth weight and GA are very similar across the countries, except in the Netherlands, where the lowest GA and weight classes represent much lower proportions (1.7% for <25 weeks GA and 0.4% for <1500 g birthweight) in comparison to other countries (ranging from 3.9 to 8.7% for <25 weeks GA and 0.9 to 3.1% for <1500 g birthweight). The Italian data show the highest mean age of mothers at delivery (33.2 years), with more than two out of five mothers older than 34 years. The Dutch population has a low proportion of multiple births, 9.9%, compared to 25.4% to 33.5% in the other countries—and infants delivered by caesarean section, 35.3%, in comparison to 66.3% to 77.8% for the other countries.

Crude mortality at 30 days and one year are further presented by GA in Tables 3 and 4, showing as expected that a GA at birth of 26 weeks or earlier is related to a significantly higher risk of mortality compared to infants born after the 26^th^ week of GA.

**Table 3:** Unadjusted mortality rates (%) within 30 days: by gestational age.

Gestational Age	Finland	Hungary	Italy	Netherlands	Norway	Scotland^b^	Sweden
< 25 weeks	62.7	73.9	75.0	83.3	59.6	52.6	43.8
25–26 weeks	22.0	34.2	36.7	36.3	13.6	13.1	14.4
27–28 weeks	11.4	13.7	13.0	13.3	7.0	4.1	6.7
29–30 weeks	3.3	5.7	5.4	8.0	4.2	1.8	2.5
31–32 weeks	1.0	3.1	3,0	3.5	1.6	0.5	1.8
> 32 weeks	0.0	6.1	2.1	NA^a^	6.4	0.5	3.4
N	1455	3562	1265	2628	1311	2015	3137

^a^ For the >32 weeks category, figs for the Netherlands were too small to be reported for two of the gestational age groupings.

^b^ Only linkable infants included.

**Table 4:** Unadjusted mortality rates (%) within 365 days: by gestational age.

Gestational Age	Finland	Hungary	Italy	Netherlands	Norway	Scotland^b^	Sweden
< 25 weeks	65.9	77.7	77.8	85.4	63.3	59.0	48.8
25–26 weeks	24.9	40.0	42.2	40.3	17.4	18.8	17.2
27–28 weeks	13.9	16.0	13.5	16.2	8.2	6.2	7.6
29–30 weeks	3.5	8.0	5.6	9.2	5.2	1.4	3.3
31–32 weeks	1.8	3.9	3.6	4.1	2.2	1.4	2.5
> 32 weeks	2.1	7.7	2.8	NA^a^	7.7	1.6	4.1
N	1455	3562	1265	2628	1311	2015	3137

^a^ For the >32 weeks category, figs for the Netherlands were too small to be reported for two of the gestational age groupings.

^b^ Only linkable infants included.

**Fig 1:**
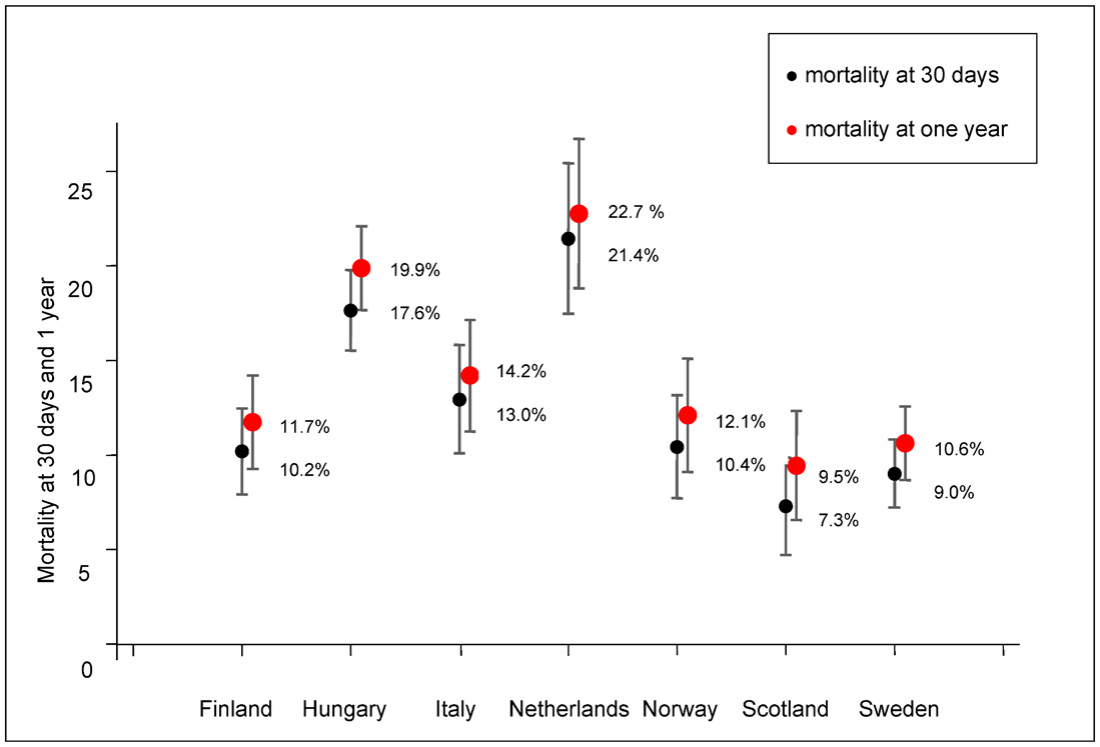
Risk adjusted mortality rates at 30 days and at 1 year. ^a^Adjusted for gestational age (GA), sex, intrauterine growth (small for gestational age), Apgar score at five minutes, parity and multiple births. A confidence interval: 95%.

However, it is interesting to note the apparent positive performance delta for Scotland and the Nordic countries (Finland, Sweden and Norway) in crude 30-day and one year mortality rates, especially for infants born before the 27^th^ week of GA, as compared to Italy, Netherlands and Hungary: proportions are on average between 10 and 20 percentage points lower for <25 weeks GA and 25–26 weeks GA. The adjusted mortality rate for all VLBW and VLGA infants after one year of follow-up was 9.5% in Scotland, 10.6% in Sweden, 11.7% in Finland, and 12.1% in Norway. Higher rates were observed in Italy (14.2%) and mainly in Hungary (19.9%) and the Netherlands (22.7%) (Fig 1).

Differences among countries were observed in the average LoS, adjusted for GA, sex, intrauterine growth, Apgar score at five minutes, parity and multiple births. As shown in both the Tables 5 and 6, these differences can be observed among both infants who survived and those who had died by the end of their first year of life. The unadjusted mean LoS for the FHE and for the first year of follow-up by GA for survivors and non-survivors are provided separately as supporting information documents (S1 and S2 Figs).

**Table 5:** Risk adjusted average number of hospital days during FHE.^a^

	Survivors	Non-survivors
Finland	60.5 (59.2–61.8)	14.3 (8.6–20.1)
Hungary	55.6 (54.8–56.4)	11.5 (9.7–13.2)
Italy	46.2 (44.5–47.8)	12.8 (9.5–16.1)
Netherlands	53.4 (52.5–54.2)	8.7 (7.3–10.2)
Norway	53.3 (52.1–54.4)	13.2 (7.7–18.7)
Scotland	50.7 (49.6–51.8)	19.9 (14.6–25.3)
Sweden	61.0 (60.0–62.0)	15.9 (9.5–22.4)

A confidence interval: 95%.

^a^ The first hospital episode (FHE) starts at the day of birth and includes all continuous hospital days, including transfers between different hospitals.

**Table 6:** Risk adjusted average number of hospital days during the first year of life.^a^

	Survivors	Non-survivors
Finland	75.2 (73.6–76.8)	23.8 (14.1–33.5)
Hungary	81.3 (80.1–82.6)	13.1 (11.1–15.1)
Italy	71.1 (67.6–74.5)	13.4 (10.0–16.8)
Netherlands	58.1 (57.1–59.1)	9.9 (8.3–11.6)
Norway	62.9 (61.4–64.4)	18.4 (10.3–26.6)
Scotland	56.8 (55.2–58.4)	26.2 (19.0–33.4)
Sweden	65.5 (64.4–66.6)	18.7 (10.8–26.5)

A confidence interval: 95%.

^a^ Figs include all hospital days, not necessarily continuous.

In survivors, the longest adjusted mean LoS during the first year of life were observed in Hungary (81.3 days) and Finland (75.2 days). The shortest adjusted average LoS during the first year among survivors was in the Netherlands (58.1 days) and Scotland (56.8 days). Non-survivors spent more time in hospital in Scotland (26.2 days) and Finland (23.8 days) compared to other countries. The shortest adjusted mean LoS for deceased infants during the first year was observed in the Netherlands (11.5 days), Hungary (13.1 days) and Italy (13.4 days). As described above, the figs for Sweden, Norway and Scotland for length of stay were measured with datasets which likely represent a healthier cohort and should therefore be interpreted with caution.

## Discussion

The incidence figs observed for VLBW and VLGA infants in the EuroHOPE study were consistent with previous literature [1, 13, 17–26] for most countries, suggesting the feasibility of linking population-wide register data among European countries and the reliability of results on incidence. Small differences between earlier studies and this study might be explained by different inclusion and exclusion criteria, by relatively low linkage rates in some countries, by differing observational periods, geographical areas and, particularly by the selective sampling in some of the previous studies focused exclusively on NICUs. A low linkage rate may explain the lower proportion of Hungarian infants enrolled in this study (1.21%), notwithstanding the broader definition in EuroHOPE compared to the Central Statistical Office [19] data (1.48%), which focused only on VLBW infants. The slight difference in the previous Finnish study (0.9% vs. 0.82% here) [18] and Norwegian data (1.04% vs. 1.22% here) might stem from the different definitions for lethal malformations we employed for data comparability and standardisation across countries. The incidence found in the nationwide EXPRESS study in Sweden [1] of extremely preterm (below 27 weeks GA), live-born infants was 0.23% for the period 2004–2007, which compares to 0.20% incidence found in EuroHOPE data. To our knowledge there have been no comparable data studies on incidence for the Netherlands and Scotland; however, surveillance data was comparable for Scotland but was considerably higher than the proportions in the EuroHOPE database for the Netherlands (Table 1).

Our study suggests better performance of Nordic countries compared to Hungary, Italy and the Netherlands, as reported in other studies [18, 19, 24]. We report a higher crude 1-year mortality rate in the Netherlands (13.1%) compared to an earlier study (10.9%) on infants born between 2002 and 2006 in a single high-level regional hospital with NICU capabilities, excluding infants born before 25 weeks GA [25]. Further explanation for high mortality in the Netherlands in EuroHOPE data may stem from customs and policies common to the country: for instance, the 2007 study describes national guidelines regarding care recommendations for extremely premature infants in which active care is advised only for infants > = 25 weeks GA and palliative care for those < 25 weeks GA. Moreover, higher mortality of Dutch infants can be related to data linkage problems and to risk adjustment procedure; the crude mortality rates are more comparable to other countries than the adjusted ones. As explained in the “Statistical analysis” section, the model for risk adjustment was based only on data from Finland, Hungary and Italy because the restriction for data use did not allow to pool data for risk adjustment modelling from all the seven countries. It should be noted that the Dutch Health Care Performance Report 2014 [27] confirmed consistently higher fetal and neonatal mortality rates in the Netherlands between 2004 and 2010 in comparison to other Western European nations (12^th^ out of 13 for neonatal mortality), though rates have been decreasing. Compared to other European nations, high percentages (almost 30% in 2012) of children are born outside of a hospital maternity unit, many at home (15.9% in 2012), in outpatient departments supervised primarily by midwives (12.5% in 2012) or in a birth center (1.5% in 2012) [28].

Regarding the differences with previous Italian data, the adjusted one-year mortality rate in several regions of Central Italy amounted to 19.3% [20], compared to 14.2% in EuroHOPE. However, the EuroHOPE study refers only to the prevalently urban Province of Rome, compared with the four regions of Central Italy in the previous study in 2001 of live births of infants below 1500 g and not necessarily all those born before 32 weeks. Finally, the previous study measured only in-hospital mortality and only infants hospitalized in NICUs.

According to the Swedish MBR 2011 report, the number of children born before GA 33^rd^ week amounted to 3979 during the period 2006–2008, compared with 3229 VLBW and VLGA infants (before exclusions) in the EuroHOPE study that, however, did not include infant of 32 GA. Their neonatal (within 28 days) mortality amounted to 7.1% of live births, compared to 8.0% here. [24] Of live-born infants in the EXPRESS study of very preterm infants (below 27 weeks GA), the crude mortality rate at 28 days was 26%, compared to 26.2% at 30 days in EuroHOPE.

The Scottish Perinatal Infant Mortality and Morbidity Report [13] reports the mortality rate within four weeks for normally-formed *singleton* neonates born at <1500 g as 12.5% for 2006, 17.5% for 2007, and 10.9% for 2008, excluding multiple births, which are reported separately and without weight or GA classifications as were used for EuroHOPE analyses. In EuroHOPE data multiple births represent 26% of the cases for Scotland. The 30-day mortality rate calculated in our data was significantly lower: only 4.7% for crude mortality and 7.3% for adjusted mortality.

The individual characteristics of the missing cases in the Scottish EuroHOPE dataset such as Apgar scores, GA and birthweight compare unfavourably with the rest of the study population and suggest that the infants that are missing from the linkable database have a poorer prognosis. As a consequence mortality among Scottish infants is very likely under-estimated in this paper and data linkage must be improved before appropriate comparisons can be made with other countries’ data.

Comparing the mortality figs with previous studies, it is likely that the EuroHOPE results provide a relevant estimate of the existing situation. Further research should enhance a more detailed understanding of mortality differences within and across countries. Among possible explanations are the centralization of care, capacity to guarantee necessary transfers between different levels of care, the quality of the prenatal care and a capacity to reduce risks related to prematurity. Furthermore, the differences can be also explained by underlying socio-economic factors such as per capita GDP, unemployment rates, poverty and population density. Last but not least, poorer outcomes can be associated with cultural patterns that may affect risky maternal behaviour, such as smoking or alcohol consumption.

As discussed at length above, there were several limitations to our study. Problems included ID attribution at birth which precluded linkage of MBR with other registers, in particular in Norway and Sweden, a problem described elsewhere [29], and in Scotland and the Netherlands. The comparability of results for the Swedish and Norwegian population in terms of LoS, and the Netherlands and Scotland for mortality is therefore limited. Furthermore, the MBR registry is not complete in Hungary and does not always include all live-born infants, though it reached 98.4% by 2008 [19]. Although the possibility to link registries in the Province of Rome increased to 95.1% by 2008 (Department of Epidemiology of the Regional Health Service—Lazio, email correspondence 21 March 2011), first hospital admissions of approximately 10% of infants transferred soon after birth were not tracked due to a late incomplete ID assignation, which reduced FHE, and consequently LoS, figs for Italy. However, it had no impact on mortality rates. In addition, the need for standardisation for the comparative objectives did not allow us to develop more sophisticated techniques for risk adjustments that could take into account a broader range of confounding factors. Differences in data collection across countries made it impossible to adjust the data for the socio-economic status of the family. Another limitation is the exclusion of stillborn infants. There might be differences among countries in defining a live-born infant [30] as suggested by the exceptionally low number of live-born infants below 25 weeks in the Netherlands.

## Conclusions

The EuroHOPE population-based study covering a large geographical area of Europe suggests that there are marked variations in mortality and average LoS between countries. In comparison to previous research, this study is not limited to a selective sample of hospitals with neonatal intensive care units but takes into consideration all hospitals in different countries and therefore the overall organization of care. Days spent in hospital care for either survivors or non-survivors were not sufficient to explain the differences in mortality between countries. Further studies are required to determine the relationship between organization of care, treatment and outcomes. European policy makers need to ensure that reliable data are available to provide information regarding which models of care and therapeutic strategies lead to improved results and cost effective resource utilization. The EuroHOPE study can be used to facilitate data gathering and standardization across countries to design methods for regular monitoring and guide efforts to improve quality and access. Policy makers should implement processes and guidelines ensuring the attribution of unique ID numbers immediately upon birth to all live-born infants to facilitate a linkage of different administrative registers, within the confines of privacy-related national legislation. Furthermore, a possibility to share confidentially anonymised data at the international level would allow for more robust risk adjustment models.

## Supporting Information

S1 Fig Unadjusted mean number of hospital days for FHE^a^ and first year of follow-up^b^: Survivors^c^. ^a^ The first hospital episode (FHE) starts the day of birth and includes all continuous inpatient hospital days, including transfers between hospitals, until discharge to home. ^b^ All hospital days during the first year, not necessarily continuous. ^c^ Infants born 2006–2008, 2006–2007 for the Netherlands, 2008–2009 for Norway. Note: too few cases precluded reporting values for infants <25 weeks GA in the Netherlands. ^d^ It is believed that the problematic linkage of follow-up admissions in 10% of Italian infants caused that the LoS figs in two groups of infants with the lowest GA (<25 weeks, 25–26 weeks) do not represent the real picture.
(TIFF)

S2 Fig Unadjusted mean number of hospital days for FHE ^a^ and first year of follow-up^b^: Non-survivors^c^. ^a^ The first hospital episode starts at the day of birth and includes all continuous hospital days, including transfers between different hospitals until discharge to home. ^b^ Figs include all hospital days during the first year, not necessarily continuous. ^c^ Infants born 2006–2008, 2006–2007 for the Netherlands, 2008–2009 for Norway.
(TIFF)

S1 Table Administrative data sources and conditions restricting the use of data. (DOCX)

S2 Table Characteristics of VLBW and VLGA infants and mothers and unadjusted mortality. Linkable infants for LoS calculation included only. ^a^ 2008–2009 for Norway
(DOCX)

S3 Table Distribution of VLBW and VLGA infants according to GA and weight at birth. ^a^ Numbers of infants are slightly lower for birth weight classes with respect to gestational age classes due to missing data on weight for some infants
(DOCX)
