# Correction: Mortality and Length of Stay of Very Low Birth Weight and Very Preterm Infants: A EuroHOPE Study

**DOI:** 10.1371/journal.pone.0177040

**Published:** 2017-05-01

**Authors:** Dino Numerato, Giovanni Fattore, Fabrizio Tediosi, Rinaldo Zanini, Mikko Peltola, Helen Banks, Péter Mihalicza, Liisa Lehtonen, Sofia Sveréus, Richard Heijink, Søren Toksvig Klitkou, Eilidh Fletcher, Amber A. van der Heijden, Fredrik Lundberg, Eelco Over, Unto Häkkinen, Timo T. Seppälä

The 13th author’s name is incorrect in the published article. The correct name is: Amber A. van der Heijden.
