# Supplementary figures and images for: Mortality and Length of Stay of Very Low Birth Weight and Very Preterm Infants: A EuroHOPE Study
Source: PLoS One. 2015 Jun 29;10(6):e0131685. doi: 10.1371/journal.pone.0131685 (PMC4488246; doi:10.1371/journal.pone.0131685)

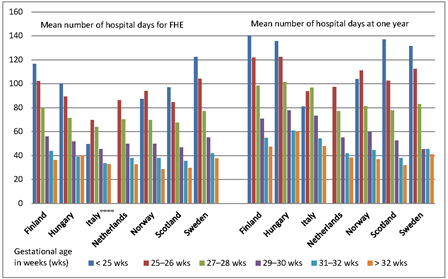

Supplement: S1 Fig — a The first hospital episode (FHE) starts the day of birth and includes all continuous inpatient hospital days, including transfers between hospitals, until discharge to home. b All hospital days during the first year, not necessarily continuous. c Infants born 2006–2008, 2006–2007 for the Netherlands, 2008–2009 for Norway. Note: too few cases precluded reporting values for infants <25 weeks GA in the Netherlands. d It is believed that the problematic linkage of follow-up admissions in 10% of Italian infants caused that the LoS figs in two groups of infants with the lowest GA (<25 weeks, 25–26 weeks) do not represent the real picture. (TIFF) [file pone.0131685.s001.tiff]

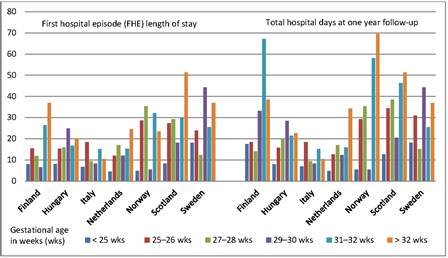

Supplement: S2 Fig — a The first hospital episode starts at the day of birth and includes all continuous hospital days, including transfers between different hospitals until discharge to home. b Figs include all hospital days during the first year, not necessarily continuous. c Infants born 2006–2008, 2006–2007 for the Netherlands, 2008–2009 for Norway. (TIFF) [file pone.0131685.s002.tiff]
