# Supplementary material for: Mortality and Length of Stay of Very Low Birth Weight and Very Preterm Infants: A EuroHOPE Study
Source: PLoS One. 2015 Jun 29;10(6):e0131685. doi: 10.1371/journal.pone.0131685 (PMC4488246; doi:10.1371/journal.pone.0131685)
Supplement: S1 Table — (DOCX) [file pone.0131685.s003.docx]

**S1 Table. Administrative data sources and conditions restricting the use of data**

|  | **Name of Organization that provided the data** | **Contact information** | **Restrictions** |
| --- | --- | --- | --- |
| Finland | National Institute for Health and Welfare (THL) | Service telephone for research authorisation applications, tel. +358 29 524 6677. | The original administrative data are confidential. The National Institute for Health and Welfare (THL) can, on a case-by-case basis, grant permission to use the registers for purposes of scientific research |
| Hungary | National Healthcare Service Center | Peter Mihalicza, [mihalicza.peter@aeek.hu](mailto:mihalicza.peter@aeek.hu) | Research access to the administrative data is possible on a case-by-case basis with a contract between the provider and the requester and requires a physical presence to access it. |
| Italy | Department of Epidemiology of the Regional Health Service – Lazio | Adele Lallo, [a.lallo@deplazio.it](mailto:a.lallo@deplazio.it) | A formal request to request the data has to be made, including the research protocol. The request is assessed on a case-by-case basis and in case of successful evaluation, the data is provided. |
| Netherlands | Statistics Netherlands, Dutch Hospital Data and The Netherlands Perinatal Registry | [cvb@cbs.nl](mailto:cvb@cbs.nl) (Statistics Netherlands), [loket@hospitaldata.eu](mailto:loket@hospitaldata.eu) (Dutch Hospital Data), Ger de Winter - [gdewinter@perinatreg.nl](mailto:gdewinter@perinatreg.nl) (Perinatal Registry) | Remote access facility, set-up by Statistics Netherlands, which is only available in research institutions in the Netherlands, was used. Interested readers should contact the institutions to ask for the possibilities. |
| Norway | The Norwegian Institute of Public Health and The Norwegian Directorate of Health | Online Registration at http://www.fhi.no/artikler/?id=69867 and http://www.fhi.no/artikler/?id=104412 | The linkage from different registries requires an approval to process personally identifiable information and dispensation from secrecy requirements for each of the data sources. |
| Scotland | Information Services Division, NHS National Services Scotland | [nss.csd@nhs.net](mailto:nss.csd@nhs.net) | Data is confidential and can only be accessed subject to obtaining all necessary ethical and regulatory approvals, but may not be available to researchers out the UK. Access to health data held by NHS Scotland, ISD or National Records Scotland - either at individual level or to link to other datasets - require an application to the Public Benefit and Privacy Panel.  The linked, patient-level data can then only be accessed via the NHS National Safe Haven. |
| Sweden | National Board of Health and Welfare | [registerservice@socialstyrelsen.se](mailto:registerservice@socialstyrelsen.se) | Following ethical approval, the National Board of Health and Welfare can on a case-by-case basis grant permission to use the registers for scientific research purposes. |
