# Supplementary material for: Mortality and Length of Stay of Very Low Birth Weight and Very Preterm Infants: A EuroHOPE Study
Source: PLoS One. 2015 Jun 29;10(6):e0131685. doi: 10.1371/journal.pone.0131685 (PMC4488246; doi:10.1371/journal.pone.0131685)
Supplement: S2 Table — Linkable infants for LoS calculation included only. a 2008–2009 for Norway (DOCX) [file pone.0131685.s004.docx]

**S2 Table. Characteristics of VLBW and VLGA infants and mothers and unadjusted mortality.**

|  | **Norway** | **Scotland** | **Sweden** |
| --- | --- | --- | --- |
| Number of VLBW and VLGA infants born in 2006-2008^a^ | 924 | 2090 | 1859 |
| Number of VLBW and VLGA infants, after exclusions | 875 | 2015 | 1812 |
| Mean (SD) gestational age, in weeks | 29.3 (2.5) | 29.5 (2.6) | 29.2 (2.4) |
| Birth weight, in grams, mean (SD) | 1260 (362) | 1282 (350) | 1301 (386) |
| Number (%) of infants by GA < 25 weeks | 45 (5.1) | 78 (3.9) | 92 (5.1) |
| Number (%) of infants by GA 25–26 weeks | 73 (8.3) | 191 (9.5) | 186 (10.3) |
| Number (%) of infants by GA 27–28 weeks | 151 (17.3) | 370 (18.4) | 326 (18.0) |
| Number (%) of infants by GA 29–30 weeks | 297 (33.9) | 625 (31.0) | 596 (32.9) |
| Number (%) of infants by GA 31–32 weeks | 254 (29.0) | 565 (28.0) | 513 (28.3) |
| Number (%) of infants by GA > 32 weeks | 55 (6.3) | 186 (9.2) | 99 (5.5) |
| N (%) infants by birth weight, < 500 grams | 8 (0.9) | 19 (1.0) | 19 (1.1) |
| N (%) infants by birth weight, 500–749 grams | 79 (9.0) | 116 (5.9) | 148 (8.2) |
| N (%) infants by birth weight, 750–999 grams | 120 (13.7) | 288 (14.6) | 240 (13.3) |
| N (%) infants by birth weight, 1000–1249 grams | 202 (23.1) | 463 (23.4) | 372 (20.7) |
| N (%) infants by birth weight,1250–1499 grams | 246 (28.1) | 637 (32.2) | 519 (28.9) |
| N (%) infants by birth weight, > 1500 grams | 215 (24.6) | 453 (22.9) | 500 (27.8) |
| Female gender (%) | 43.9 | 46.6 | 42.5 |
| Apgar score at five minutes, median | 9 | 9 | 9 |
| Appropriate for gestational age (%) | 78.1 | 77.2 | 80.3 |
| Small for gestational age (%) | 4.6 | 5.6 | 6.1 |
| Multiple birth (%) | 27.3 | 26.0 | 25.4 |
| First delivery (%) | 54.5 | 58.9 | 82.6 |
| Ceasarean delivery (%) | 67.0 | 59.4 | 66.4 |
| Malformations, number (%) | 113 (12.9) | 269 (13.3) | 125 (6.9) |
| Mother’s characteristics, age in years (SD) | NA | 29.0 (6.6) | NA |
| Mothers over 34 years (%) | NA | 23.5 | NA |
| Unadjusted 30 day crude mortality (%) | 4.2 | 4.7 | 2.3 |
| Unadjusted 1 year crude mortality (%) | 5.5 | 6.3 | 3.1 |

Linkable infants for LoS calculation included only.

^a^ 2008-2009 for Norway
