# Supplementary material for: Mortality and Length of Stay of Very Low Birth Weight and Very Preterm Infants: A EuroHOPE Study
Source: PLoS One. 2015 Jun 29;10(6):e0131685. doi: 10.1371/journal.pone.0131685 (PMC4488246; doi:10.1371/journal.pone.0131685)
Supplement: S3 Table — a Numbers of infants are slightly lower for birth weight classes with respect to gestational age classes due to missing data on weight for some infants (DOCX) [file pone.0131685.s005.docx]

**S3 Table. Distribution of VLBW and VLGA infants according to GA and weight at birth.**

|  | **Finland** | **Hungary** | **Italy** | **Netherlands** | **Norway** | **Scotland** | **Sweden** |
| --- | --- | --- | --- | --- | --- | --- | --- |
| Number (%) of infants by GA < 25 weeks | 126 (8.7) | 291 (8.2) | 72 (5.7) | 48 (1.8) | 109 (8.3) | 149 (6.4) | 260 (8.3) |
| Number (%) of infants by GA 25–26 weeks | 177 (12.2) | 442 (12.4) | 128 (10.1) | 273 (10.4) | 132 (10.1) | 247 (10.6) | 389 (12.4) |
| Number (%) of infants by GA 27–28 weeks | 273 (18.8) | 649 (18.2) | 207 (16.4) | 481 (18.3) | 243 (18.5) | 417 (17.9) | 582 (18.6) |
| Number (%) of infants by GA 29–30 weeks | 397 (27.3) | 924 (25.9) | 354 (28.0) | 752 (28.6) | 385 (29.4) | 692 (29.7) | 927 (29.6) |
| Number (%) of infants by GA 31–32 weeks | 384 (26.4) | 877 (24.6) | 363 (28.7) | 804 (30.6) | 364 (27.8) | 616 (26.4) | 834 (26.6) |
| Number (%) of infants by GA > 32 weeks | 97 (6.7) | 379 (10.6) | 141 (11.1) | 270 (10.3) | 78 (5.9) | 209 (9.0) | 145 (4.6) |
| Number (%) of infants by birth weight,^a^ < 500 grams | 44 (3.1) | 90 (2.6) | 28 (2.3) | <20(<0.8) | 23 (1.8) | 40 (1.7) | 57 (1.8) |
| Number (%) of infants by birth weight, 500–749 grams | 155 (10.8) | 404 (1153) | 133 (10.8) | 190 (7.4) | 158 (12.1) | 181 (7.9) | 380 (12.2) |
| Number (%)of infants by birth weight, 750–999 grams | 222 (15.4) | 774 (21.9) | 184 (14.9) | 420 (16.3) | 198 (15.1) | 351 (15.3) | 481 (15.5) |
| Number (%)of infants by birth weight, 1000–1249 grams | 309 (21.5) | 550 (15.6) | 266 (21.6) | 594 (23.1) | 279 (21.3) | 523 (22.8) | 612 (19.7) |
| Number (%)of infants by birth weight,1250–1499 grams | 415 (28.9) | 1109 (31.4) | 343 (27.8) | 731 (28.4) | 340 (25.9) | 693 (30.3) | 786 (25.2) |
| Number (%)of infants by birth weight, > 1500 grams | 293 (20.4) | 601 (17.0) | 279 (22.6) | 630 (23.5) | 307 (23.4) | 502 (21.9) | 797 (25.6) |

^a^Numbers of infants are slightly lower for birth weight classes with respect to gestational age classes due to missing data on weight for some infants
